# Supplementary figures and images for: Pooling samples to increase testing capacity with Xpert Xpress SARS-CoV-2 during the Covid-19 pandemic in Lao People’s Democratic Republic
Source: PLoS One. 2022 Sep 29;17(9):e0275294. doi: 10.1371/journal.pone.0275294 (PMC9522287; doi:10.1371/journal.pone.0275294)

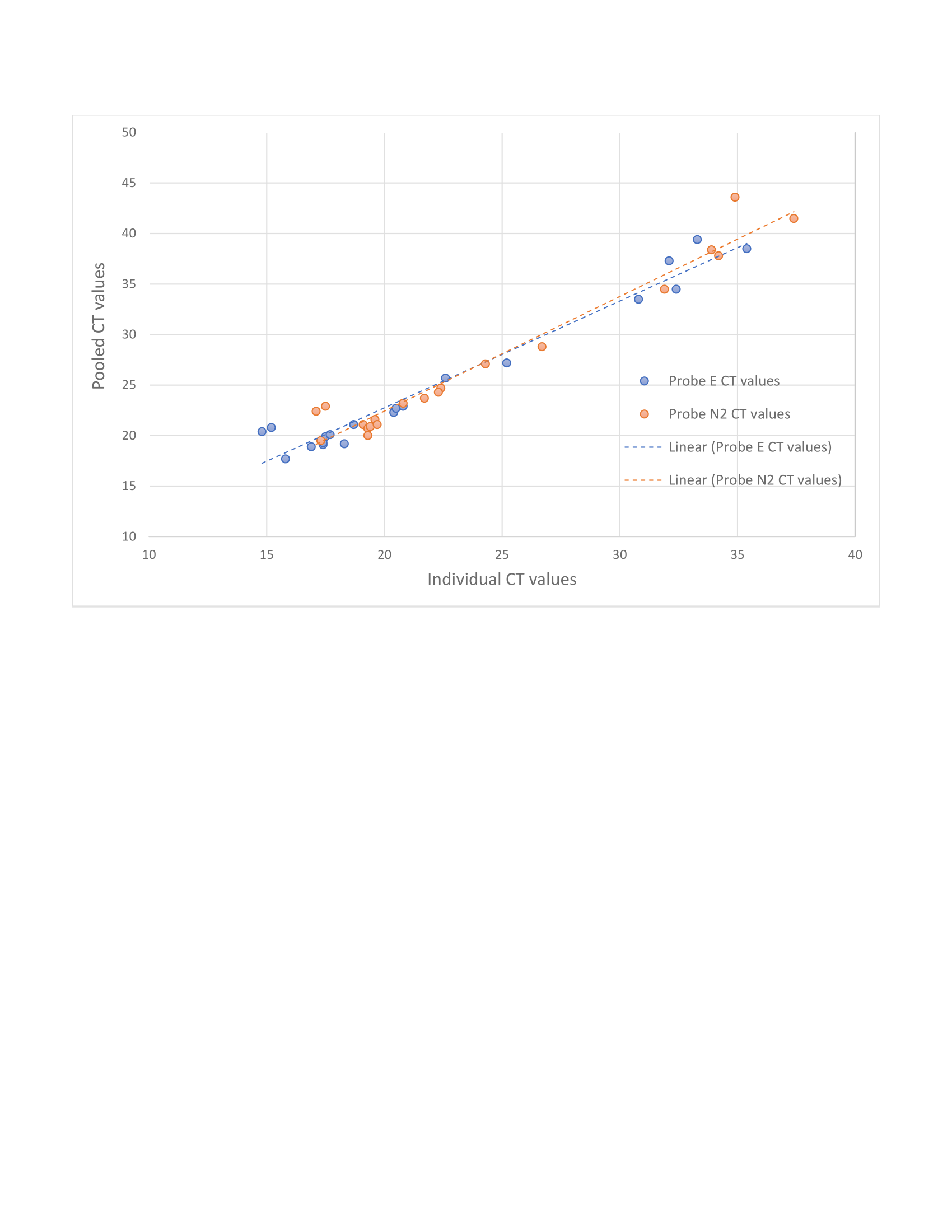

Supplement: S1 Fig — (TIF) [file pone.0275294.s001.tif]

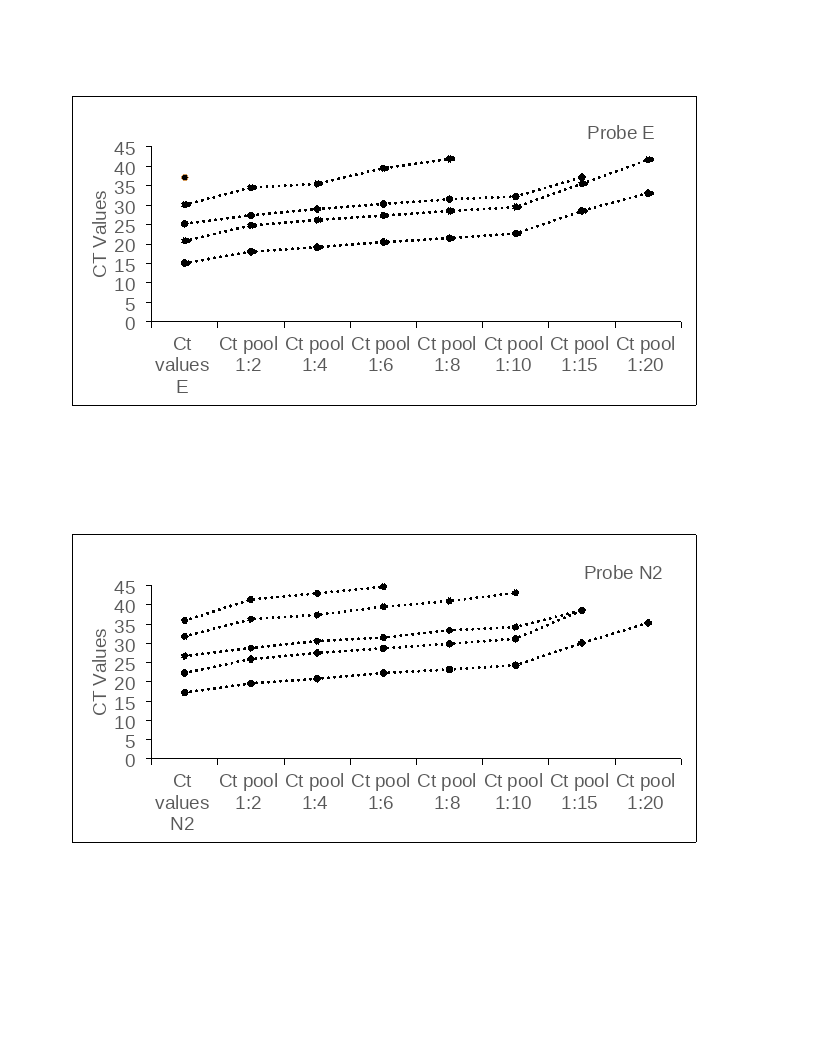

Supplement: S2 Fig — (TIFF) [file pone.0275294.s002.tiff]
