# Supplementary material for: Pooling samples to increase testing capacity with Xpert Xpress SARS-CoV-2 during the Covid-19 pandemic in Lao People’s Democratic Republic
Source: PLoS One. 2022 Sep 29;17(9):e0275294. doi: 10.1371/journal.pone.0275294 (PMC9522287; doi:10.1371/journal.pone.0275294)
Supplement: S1 Table — (DOCX) [file pone.0275294.s003.docx]

S1 Table. CT values for the probes E and N2 for both individual and pooled results

| Number of positive samples included in Xpress-positive pools | Individual CT values | | Pooled CT values | |
| --- | --- | --- | --- | --- |
|  | E | N2 | E | N2 |
| 0 | 0 | 0 | 0 | 43.1 |
|  | 0 | 0 | 0 | 44.8 |
| 1 | 15.8 | 17.3 | 17.7 | 19.5 |
|  | 16.9 | 19.1 | 18.9 | 21.1 |
|  | 17.4 | 19.3 | 19.1 | 20.7 |
|  | 18.3 | 19.3 | 19.2 | 20 |
|  | 17.4 | 19.4 | 19.3 | 20.9 |
|  | 17.5 | 19.6 | 19.9 | 21.6 |
|  | 17.7 | 19.7 | 20.1 | 21.1 |
|  | 14.8 | 17.1 | 20.4 | 22.4 |
|  | 15.2 | 17.5 | 20.8 | 22.9 |
|  | 18.7 | 20.8 | 21.1 | 23.2 |
|  | 20.4 | 21.7 | 22.3 | 23.7 |
|  | 20.5 | 22.4 | 22.7 | 24.7 |
|  | 20.8 | 22.3 | 22.9 | 24.3 |
|  | 22.6 | 24.3 | 25.7 | 27.1 |
|  | 25.2 | 26.7 | 27.2 | 28.8 |
|  | 30.8 | 31.9 | 33.5 | 34.5 |
|  | 32.4 | 34.2 | 34.5 | 37.8 |
|  | 32.1 | 33.9 | 37.3 | 38.4 |
|  | 35.4 | 37.4 | 38.5 | 41.5 |
|  | 33.3 | 34.9 | 39.4 | 43.6 |
| 2 | 21.7 | 23.3 | 17.2 | 19.2 |
|  | 15.1 | 17.2 |  |  |
|  | 19.5 | 20.7 | 17.8 | 19.6 |
|  | 16.1 | 17.5 |  |  |
|  | 39.9 | 41.7 | 19.1 | 20.4 |
|  | 17.5 | 19.2 |  |  |
|  | 17.6 | 18.9 | 20.3 | 21.5 |
|  | 23.8 | 25.1 |  |  |
|  | 26.4 | 27.8 | 28.2 | 29.4 |
|  | 35.7 | 36.2 |  |  |
|  | 34.5 | 36.3 | 32.9 | 35.2 |
|  | 32.3 | 33.6 |  |  |
| 3 | 37.1 | 35.9 | 31.7 | 33.6 |
|  | 30.1 | 31.8 |  |  |
|  | 33.3 | 34.5 |  |  |
|  | 33.3 | 33.9 | 33 | 33.7 |
|  | 31.3 | 32.3 |  |  |
|  | 34.9 | 37.1 |  |  |
| 4 | 37.7 | 41.1 | 36.1 | 41.7 |
|  | 38.2 | 41.9 |  |  |
|  | 31.9 | 34.2 |  |  |
|  | 36.4 | 38.6 |  |  |
