# Supplementary material for: Pooling samples to increase testing capacity with Xpert Xpress SARS-CoV-2 during the Covid-19 pandemic in Lao People’s Democratic Republic
Source: PLoS One. 2022 Sep 29;17(9):e0275294. doi: 10.1371/journal.pone.0275294 (PMC9522287; doi:10.1371/journal.pone.0275294)
Supplement: S2 Table — (DOCX) [file pone.0275294.s004.docx]

S2_Table. Effect of dilution on CT values of positive samples

|  | Individual | 1:2 | 1:4 | 1:6 | 1:8 | 1:10 | 1:15 | 1:20 |
| --- | --- | --- | --- | --- | --- | --- | --- | --- |
| Probe E | **CT value** | | | | | | | |
| Sample 1 | 15.1 | 18 | 19.2 | 20.5 | 21.5 | 22.7 | 28.5 | 33.1 |
| Sample 2 | 20.8 | 24.8 | 26.2 | 27.3 | 28.5 | 29.5 | 35.5 | 41.7 |
| Sample 3 | 25.2 | 27.4 | 29 | 30.3 | 31.5 | 32.2 | 37.2 | NA |
| Sample 4 | 30.1 | 34.5 | 35.5 | 39.5 | 41.9 | NA | NA | NA |
| Sample 5 | 37.1 | NA | NA | NA | NA | NA | NA | NA |
| Probe N2 |  | | | | | | | |
| Sample 1 | 17.2 | 19.6 | 20.8 | 22.3 | 23.2 | 24.3 | 30.1 | 35.3 |
| Sample 2 | 22.3 | 25.9 | 27.5 | 28.7 | 29.9 | 31.2 | 38.6 | NA |
| Sample 3 | 26.7 | 28.8 | 30.6 | 31.5 | 33.4 | 34.2 | 38.6 | NA |
| Sample 4 | 31.8 | 36.3 | 37.4 | 39.5 | 41 | 43.1 | NA | NA |
| Sample 5 | 35.9 | 41.4 | 43 | 44.7 | NA | NA | NA | NA |
