# Supplementary material for: Pooling samples to increase testing capacity with Xpert Xpress SARS-CoV-2 during the Covid-19 pandemic in Lao People’s Democratic Republic
Source: PLoS One. 2022 Sep 29;17(9):e0275294. doi: 10.1371/journal.pone.0275294 (PMC9522287; doi:10.1371/journal.pone.0275294)
Supplement: Attachment — Submitted filename: Response to Reviewers_rev1.docx [file pone.0275294.s006.docx]

Response to Reviewers

1. Please ensure that your manuscript meets PLOS ONE's style requirements, including those for file naming.

Edited as suggested.

2. Please provide additional details regarding participant consent. In the ethics statement in the Methods and online submission information, please ensure that you have specified what type you obtained (for instance, written or verbal, and if verbal, how it was documented and witnessed). If your study included minors, state whether you obtained consent from parents or guardians. If the need for consent was waived by the ethics committee, please include this information.

In the manuscript line 150-153, it is stated: “Need for ethical approval and informed consent were waived by the National Center for Laboratory and Epidemiology, Lao PDR Ministry of Health. The Center is the delegated authority for Covid-19 testing. Permission was granted through the Emergency Operations Centre under Lao PDR Task force for Covid-19 Prevention, Control and Response.” Therefore, we haven’t changed the text. However, we have added the level 2 heading “Ethics statement” at the beginning of the paragraph.

The same statement is also already in the online submission form.

3. Thank you for stating the following financial disclosure. Please state what role the funders took in the study. If the funders had no role, please state: ""The funders had no role in study design, data collection and analysis, decision to publish, or preparation of the manuscript.""

If this statement is not correct you must amend it as needed.

Please include this amended Role of Funder statement in your cover letter; we will change the online submission form on your behalf.

We have removed the section line 450-452, that stated: “The sponsors had no role in the study design; collection, analysis and interpretation of data; the writing of the manuscript; the decision to submit the manuscript for publication”.

We have now included the following statement “The funders had no role in study design, data collection and analysis, decision to publish, or preparation of the manuscript” in the revised cover letter as suggested.

4. Thank you for stating the following in the Funding Section of your manuscript.

We note that you have provided funding information that is not currently declared in your Funding Statement. However, funding information should not appear in the Acknowledgments section or other areas of your manuscript. We will only publish funding information present in the Funding Statement section of the online submission form.

Please remove any funding-related text from the manuscript…

We have removed all funding-related text as suggested.

…. and let us know how you would like to update your Funding Statement. Currently, your Funding Statement reads as follows:

“This research was funded in part by the Global Fund to Fight AIDS, Tuberculosis and Malaria (LAO-T-GFMOH country grant), the Global Fund Covid-19 Response Mechanism (C19RM), the National Center for Laboratory and Epidemiology and the National Tuberculosis Control Center from the Ministry of Health of Lao PDR.

TW is supported by grants from: the Wellcome Trust, UK (209075/Z/17/Z); the Medical Research Council, Department for International Development, and Wellcome Trust, UK (Joint Global Health Trials, MR/V004832/1), the Medical Research Council, UK (MR/V028618/1); the Academy of Medical Sciences, UK; and the Swedish Health Research Council, Sweden.”

We confirm that this funding statement is correct.

Please include your amended statements within your cover letter; we will change the online submission form on your behalf.

We have edited the covert letter as suggested.

5. Thank you for stating the following in your Competing Interests section. This information should be included in your cover letter; we will change the online submission form on your behalf.

We have edited the cover letter as suggested.

6. Your ethics statement should only appear in the Methods section of your manuscript. If your ethics statement is written in any section besides the Methods, please move it to the Methods section and delete it from any other section. Please ensure that your ethics statement is included in your manuscript, as the ethics statement entered into the online submission form will not be published alongside your manuscript.

We have removed the duplicate ethical statement line 425-429. The ethical statement now only appears once at line 150-153, in the Methods section, as suggested.

7. Please review your reference list to ensure that it is complete and correct. If you have cited papers that have been retracted, please include the rationale for doing so in the manuscript text, or remove these references and replace them with relevant current references. Any changes to the reference list should be mentioned in the rebuttal letter that accompanies your revised manuscript. If you need to cite a retracted article, indicate the article’s retracted status in the References list and also include a citation and full reference for the retraction notice.

The reference list was built using endnote, and is accurate with no retracted references.

Reviewer #2: From the methodology provided, samples were collected, pooled and tested, there is a need to add information on what was used in diluting the samples. which of the samples were diluted, the entire pool or individual samples that makes up the pool?

We have added the following text to clarify methodology at line 118 as suggested by the reviewer: “Pools were created by pipetting equal amounts (200 µL) of the individual samples directly into a container with the virus transport medium (BD Universal Viral Transport System, catalogue number 220220), and mixed together into a single use 2 mL cryovial tube.”

Another statement was added at line 132: “For this bench evaluation, 200 µL from the individual positive samples with known CT values were diluted using multiples of 200 µL fresh virus transport medium to replicate the desired dilution of the pools”.

Author should give a brief description of the analysis in the methodology to specifically show what Probe E and N2 signifies. And for clarity what gene was targeted.

Probe E and N2 refer to probes targeting genes E and N2 respectively. A statement was added at line 118 to clarify the terminology as suggested: “The Xpert Xpress is specifically designed to amplify sequences of the envelope (E) and the nucleocapsid (N2) of the virus to generate tests results. If one or more SARS-CoV-2 nucleic acid targets (E, N2) has a CT within the valid range, the test reports a positive result.”

May include catalogue number/make of single viral transport media tube

The reference was added at line 119 as suggested.
